# Comparison of Repetitive Cardiac Output Measurements at Rest and End-Exercise by Direct Fick Using Pulse Oximetry vs. Blood Gases in Patients With Pulmonary Hypertension

**DOI:** 10.3389/fmed.2021.776956

**Published:** 2021-11-23

**Authors:** Milos Duknic, Mona Lichtblau, Stéphanie Saxer, Charlotte Berlier, Simon R. Schneider, Esther I. Schwarz, Arcangelo F. Carta, Michael Furian, Konrad E. Bloch, Silvia Ulrich

**Affiliations:** ^1^Department of Pulmonology, University Hospital Zürich, Zurich, Switzerland; ^2^University of Zurich, Zurich, Switzerland

**Keywords:** cardiac output, direct Fick, pulse oximetry, exercise, pulmonary hypertension

## Abstract

**Background:** Exact and simultaneous measurements of mean pulmonary artery pressure (mPAP) and cardiac output (CO) are crucial to calculate pulmonary vascular resistance (PVR), which is essential to define pulmonary hypertension (PH). Simultaneous measurements of mPAP and CO are not feasible using the direct Fick (DF) method, due to the necessity to sample blood from the catheter-tip. We evaluated a modified DF method, which allows simultaneous measurement of mPAP and CO without needing repetitive blood samples.

**Methods:** Twenty-four patients with pulmonary arterial or chronic thromboembolic PH had repetitive measurements of CO at rest and end-exercise during three phases of a crossover trial. CO was assessed by the original DF method using oxygen uptake, measured by a metabolic unit, and arterial and mixed venous oxygen saturations from co-oximetry of respective blood gases served as reference. These CO measurements were then compared with a modified DF method using pulse oximetry at the catheter- and fingertip.

**Results:** The bias among CO measurements by the two DF methods at rest was −0.26 L/min with limits of agreement of ±1.66 L/min. The percentage error was 28.6%. At the end-exercise, the bias between methods was 0.29 L/min with limits of agreement of ±1.54 L/min and percentage error of 16.1%.

**Conclusion:** Direct Fick using a catheter- and fingertip pulse oximetry (DFp) is a practicable and reliable method for assessing CO in patients with PH. This method has the advantage of allowing simultaneous measurement of PAP and CO, and frequent repetitive measurements are needed during exercise.

**Clinical Trial Registration:** https://clinicaltrials.gov/ct2/show/NCT02755259, identifier: NCT02755259.

## Introduction

In the absence of relevant lung diseases, the two major forms of precapillary pulmonary hypertension (PH) are pulmonary arterial and chronic thromboembolic pulmonary hypertension (PAH/CTEPH). PH is a relatively rare condition with potentially drastic limitations in prognosis and quality of life (1–3). PH is diagnosed and hemodynamically classified by right heart catheterization (RHC) as mean pulmonary artery pressure (mPAP) >20 mmHg with a pulmonary artery wedge pressure (PAWP) ≤ 15 mmHg (3–5). In 2019, it was proposed to include a pulmonary vascular resistance (PVR) of ≥ 3 WU into the definition of precapillary PH, to increase the specificity of diagnosis (5). To calculate the PVR, simultaneous measurement of cardiac output (CO) and mPAP is necessary (6). Thus, accurate assessment of CO is crucial for diagnosis, classification, therapeutic decisions, and prognostic evaluation in PH (5, 7). Inaccuracy and imprecision of CO measurement techniques can lead to misdiagnosis or inadequate treatment decisions. These aspects highlight the relevance of reliable CO measurements at rest, but also during vasoreactivity testing and exercise challenge at the time of diagnosis or during follow-up of patients with PH to evaluate effects of therapy and prognosis (8).

Cardiac output can be measured with different methods. The gold standard has been the direct Fick (DF) method, albeit with certain reservations because it requires simultaneous measurement of oxygen consumption and, in its original form, simultaneous arterial and mixed venous blood samples to assess oxygen saturation in the blood by co-oximetry, which limits frequent repetitive assessments (9). Furthermore, mPAP cannot be measured simultaneously when sampling blood from the catheter-tip, which prohibits simultaneous assessments of mPAP, CO, and PAWP to calculate PVR with standard equipment, such as the widely used Swan-Ganz catheter, and thus may limit accurate measurement in non-steady state condition. This challenges accurate hemodynamic measurement in a biologically fluctuating system already at rest but makes the DF method impractical during incremental exercise. Replacing repetitive measurements of arterial and mixed venous oxygen content by continuous assessment of pulse- and catheter-tip oximetry (SpO_2_ and SpmvO_2_) requires blood gas sampling only once. Additionally, it allows—together with continuous oxygen uptake (VO_2_) registration—continuous CO assessment. This slightly modified DF method is herein referred to as “DF pulse oximetry” (DFp).

Another widely used method to measure CO is intermittent pulmonary thermodilution (TD), which does not require equipment to assess VO_2_ from breathing gases.

The main aim of this study was to evaluate the precision and accuracy of CO measured by DF using a catheter and fingertip pulse oximetry (DFp) in comparison to DF using oxygen saturation derived by co-oximetry from blood gases (DF), at rest and at end-exercise in patients with PH. An additional aim was to compare TD vs. DF at rest and end-exercise.

## Methods

### Study Design and Participants

This study included patients with PAH/CTEPH diagnosed according to international guidelines from 2015 (3). Patients without PH or PH classified to other diagnostic groups were excluded. In all patients, the right heart catheterization (RHC) examination was clinically indicated, and patients provided written informed consent to participate in a prospective randomized, placebo-controlled, double-blinded, triple-phase, and crossover trial evaluating acute hemodynamic effects of acetazolamide at rest and end-exercise. The study design, methods, and results on the acute hemodynamic effect of acetazolamide on PVR and other measures at rest have been published (10). This study focuses on the comparison of the CO at rest and end-exercise measured by different methods, and these results have not been previously published.

The study complies with the declaration of Helsinki and was approved by the local ethics committee (BASEC 2016-00089, and the trial was registered at ClinicalTrials.gov (NCT02755259).

### Interventions and Assessments

#### Catheterization and CO Measurements

The right heart catheterization was performed in the supine position using a balloon-tipped, triple-lumen, fluid-filled 7.5F Swan-Ganz catheter (Swan Ganz CCOmbo V, Edwards Lifesciences, Irvine, CA, USA) inserted into the right or left internal jugular vein under sonographic guidance. Transducers were set at the midthoracic level and zeroed to atmospheric pressure (11). Additionally, A 5F Teflon catheter was inserted into a radial artery. CO using different methods (DF, DFp, and TD) was repetitively assessed at rest and at end-exercise in three phases each 60–90 min apart according to the protocol (10). Resting blood gas was sampled in the supine position, all other measurements were conducted in the semi-supine position.

#### Exercise and Spirometry

Patients performed cycling exercise in semi-supine position with 3 min stepwise incremental increase of work-rates by 10–20 Watts to maximal exhaustion (Thera-vital Ergometer; Medica Medizin GmbH). Oxygen uptake (VO_2_) was measured with a metabolic unit (Ergo-, Spirostik) and BlueCherry (Geratherm Respiratory, Germany) and averaged over 15 seconds.

#### Blood Gas Analysis and Oximetry

Arterial and mixed venous blood samples were taken from the radial artery line and the pulmonary artery catheter-tip. The first resting mixed venous blood gas analysis (SmvO_2_) was used to calibrate pulse oximetry at the catheter-tip SpmvO_2_. Finger- and catheter-tip pulse oximetry (SpO_2_ and SpmvO_2_) were continuously measured and registered in LabChart (Version 8.1.16; ADInstruments).

To calculate the CO by the DF methods, we used the following formula: DF = VO_2_ [l/min]/(hemoglobin[g/dl] × 13.4 × (SaO_2_-SmvO_2_ [%]/100) × 1,000) (12). The DF method using arterial- and mixed venous blood gases served as reference for comparison with DFp and TD. To calculate DFp, we used in analogy the formula: DFp = VO_2_ [l/min]/(hemoglobin[g/dl] × 13.4 × (SpO_2_-SpmvO_2_ [%]/100) × 1,000).

#### Intermittent TD

Cardiac output by TD was measured in triplicate by cold saline injection (Vigilance II, Edwards Lifesciences, Irvine, CA, USA) (3, 13, 14). At each time point, two to three measurements were performed. Measurements were excluded if they varied >10% from each other, and the mean value was calculated using the remaining measurements.

#### Outcomes

The main purpose was the difference in CO between DF based on blood gas analysis and DF based on pulse oximetry at rest and end-exercise. An additional purpose was the difference in CO between DF and TD at rest and end-exercise.

### Statistical Analysis

Assessed data were inspected visually in the LabChart program for plausibility, and artifacts were deleted. Continuously registered data were averaged over periods of 15 seconds. Where appropriate, i.e., during a steady state, missing data points were carried forward. The results are expressed as means ± SD unless indicated otherwise. The normality assumption was tested using the Shapiro-Wilk test. Heteroscedasticity was assessed using the studentized Breusch-Pagan test. The agreement and bias were assessed by the method described by Bland and Altman (15). Bias was expressed as the mean of the differences obtained by different techniques (e.g., oDF-DF and TD-DF) with its 95% CI. The limits of agreement are expressed as mean ± 1.96 SD. Percentage error was derived by dividing the limits of the agreement by the mean of both methods. The coefficient of variation was obtained by dividing the SD of a method by its respective mean. The repeatability coefficient was calculated according to Carstensen et al. (16) for linked or paired replicates under the assumption of identical conditions. The paired data structure also had an influence on further statistical parameters. All statistical analyses were performed using RStudio (Version 1.2.1578, R Studio Inc., San Francisco, CA, USA). Acceptance criteria were defined as limits of agreement within 2 L/min and a percentage error of 30% (17).

## Results

### Patient Characteristics

We included 24 patients (7 female) with PH (CTEPH, *n* = 17; PAH, *n* = 7). Patient characteristics and baseline resting hemodynamics are shown in Table 1. The mean age was 59 ± 14 years, body mass index (BMI) 27.9 ± 4.6 kg/m^2^, heart rate (HR) 75 ± 11 bpm, and mPAP 37 ± 12 mmHg.

**Table 1:** Baseline characteristics of the study population.

**Baseline characteristics**		**Number (%) or mean ± SD**
No. of patients		24
Sex (%)	Male	17 (71%)
	Female	7 (29%)
Age, yr		59 ± 14
BMI, kg/m^2^		27.9 ± 4.6
Pulmonary hypertension classification (%)	PAH	7 (29%)
	CTEPH	17 (71%)
NYHA (%)	I	2 (8%)
	II	15 (63%)
	III	7 (29%)
	IV	0 (0%)
6-min walk distance, m		531 ± 112
Body surface area, m^2^		1.99 ± 0.19
NT-proBNP, ng/L (median [IQR])		258 [82, 485]
Heart rate, bpm		75 ± 11
SpO_2_, % (median [IQR])		93 [91, 95]
SmvO_2_, % (median [IQR])		63 [60, 67]
Mean pulmonary artery pressure, mmHg		37 ± 11
Pulmonary artery wedge pressure, mmHg		11 ± 2
Pulmonary vascular resistance, WU		5.2 ± 2.7
Mean systemic blood pressure, mmHg		94 ± 9
Right atrial pressure, mmHg		6 ± 3

*Data are presented as mean ± SD unless indicated otherwise. BMI, Body mass index; PAH, pulmonary arterial hypertension; CTEPH, chronic thromboembolic pulmonary hypertension; NYHA, New York Heart Association (score); NT-proBNP, N-terminal pro b-type Natriuretic Peptide; WU, Woods units*.

### DF Using Blood Gases vs. DF Using Pulse Oximetry

**Figure 1:**
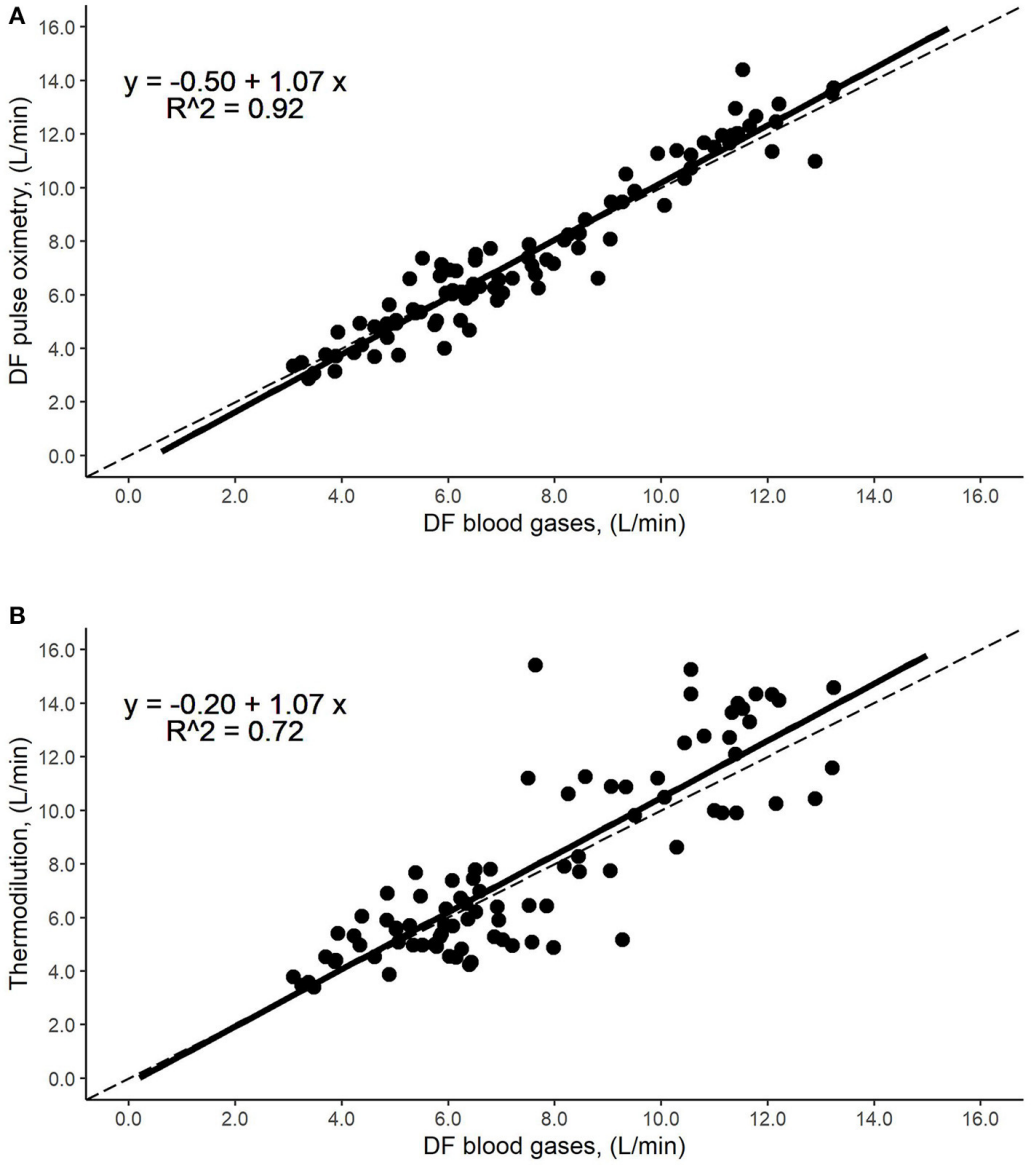
Identity plot of cardiac output (CO) measurement methods. Relationship by regression, such as data points from rest and end-exercise; the dashed line represents the line of equality. The black solid line represents the regression line, while the dots stand for the corresponding measurements. **(A)** Direct Fick using pulse oximetry (DFp) vs. direct Fick using blood gases (DF): The regression line shows an R^2^ of 0.92, a slope of 1.07 with an intercept of −0.50 L/min and no displacement over the line of identity over a broad range of CO values. **(B)** Intermittent thermodilution (TD) vs. direct Fick using blood gases (DF): The regression line shows an R^2^ of 0.72, a slope of 1.07 with an intercept of −0.20 L/min and no displacement over the line of identity over a broad range of CO values.

The average CO of both methods at rest, end-exercise, and both combined, and further statistical parameters are summarized in Table 2. The regression equation including data at rest and end-exercise had a slope of 1.07 with an intercept of −0.50 L/min and an R^2^ of 0.92 (Figure 1A). The normality assumption was met (*p* = 0.64).

**Table 2:** Mean cardiac output (CO) and statistical parameters at rest, end-exercise, and both combined.

**Cardiac output**	**Rest**	**End-exercise**	**Combined rest and end-exercise**
Maximal workload, W		80 ± 37	
CO direct Fick blood gases, L/min	5.9 ± 1.5	9.4 ± 2.6	7.5 ± 2.7
No. of measurements	49	42	91
CO direct Fick pulse oximetry, L/min	5.5 ± 1.5	10.0 ± 3.2	7.5 ± 3.3
No. of measurements	66	51	117
CO thermodilution, L/min	5.5 ± 1.2	10.7 ± 3.0	7.3 ± 3.4
No. of measurements	64	50	114
**Statistical parameters comparing cardiac output measures by direct Fick using blood gases vs. direct Fick using pulseoximetry**			
Bias (95% CI), L/min	−0.26 (−0.62 to 0.09)	0.29 (−0.08 to 0.67)	0.01 (−0.35 to 0.37)
Limits of agreement, L/min	−1.92 to 1.39	−1.25 to 1.83	−1.67 to 1.69
Percentage error, %	28.6	16.1	22.3
Coefficient of variation (DF/DFp), %	25.3^*^/25.9	27.3^*^ / 29.6	36.0/39.9
Repeatability coefficient (DF/DFp), L/min	2.03/ <0.01	0.99 / 1.72	0.01/2.71
**Statistical parameters comparing cardiac output measures by direct Fick vs. intermittent thermodilution**			
Bias (95% CI), L/min	0.45 (−0.98 to 0.08)	1.39 (0.37 to 2.42)	0.36 (−0.42 to 1.13)
Limits of agreement, L/min	−2.93 to 2.03	−2.84 to 5.62	−3.28 to 4.00
Percentage error, %	44.6	41.6	47.2
Coefficient of variation (DF/TD), %	24.7^*^/19.8	26.1^*^/26.8	36.2/43.7
Repeatability coefficient (DF/TD), L/min	2.95/1.50	2.72/1.86	0.01/4.33

*Data are presented as mean ± SD unless indicated otherwise. W: Watts; DF: direct Fick using arterial and mixed-venous blood gases; DFp, direct Fick using finger- and cathetertip pulseoximetry; TD, thermodilution.*

* *The coefficients of variation for DF vs. DFp and vs. TD do not match, because measurements were included as pairs (i.e., if a TD measurement was not available the corresponding DF measurement was not included and vice versa)*.

**Figure 2:**
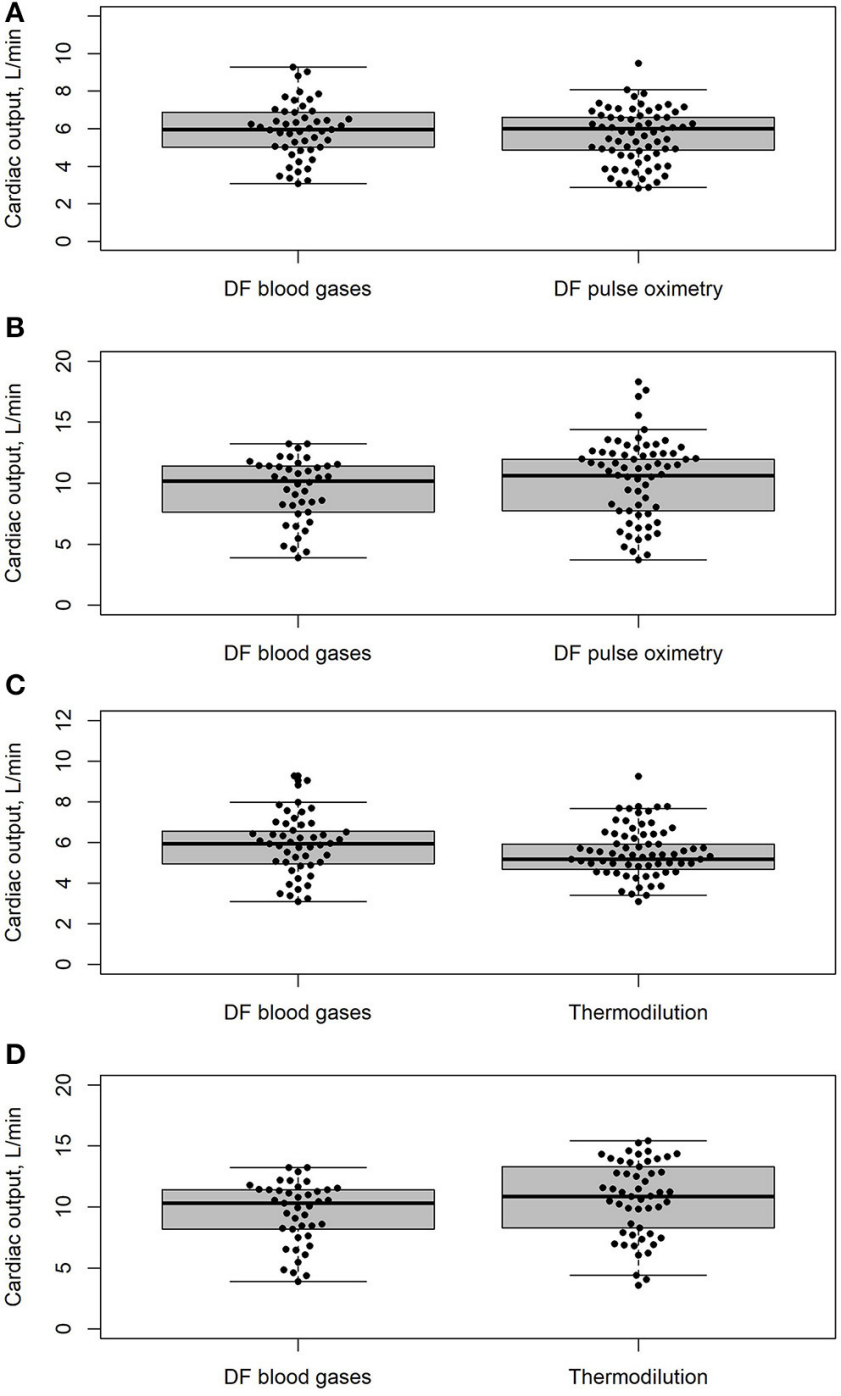
Distribution of cardiac output (CO) measurements by different measurement methods at rest and end-exercise are shown as boxplots. The horizontal line represents the median, the box the 25th to 75th percentile, the whiskers 5th to 95th percentile, and the dots represent the individual values, including outliers. **(A)** CO measurement at rest: direct Fick using pulse oximetry (DFp) vs. direct Fick using blood gases (DF)*. **(B)** CO measurements at end-exercise: direct Fick using pulse oximetry (DFp) vs. direct Fick using blood gases (DF)*. **(C)** CO measurements at rest: intermittent thermodilution (TD) vs. direct Fick using blood gases (DF)*. **(D)** CO-measurements at end-exercise: intermittent thermodilution (TD) vs. direct Fick using blood gases (DF)*. *if the corresponding CO measurement was not available or missing, both measurements were excluded.

**Figure 3:**
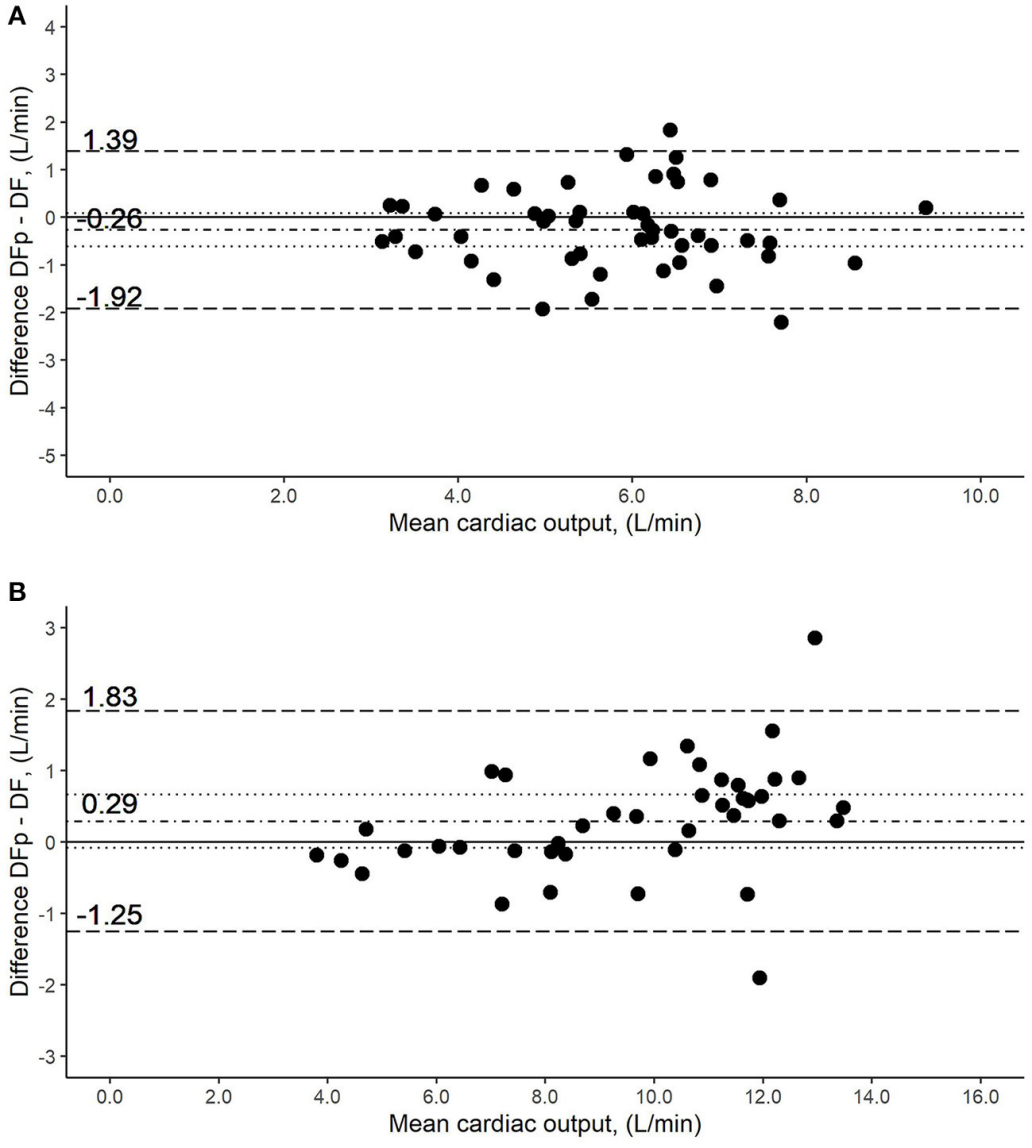
Bland-Altman plot of the comparison of cardiac output (CO) measurement methods by direct Fick using pulse oximetry (DFp) vs. direct Fick using blood gases (DF). *Y*-axis was calculated as the mean of the two methods being compared. The solid line represents no difference in the means by the two methods. The dot-dashed line around zero represents the measured difference of the means, while the dotted lines stand for the 95% CI. The broken lines above and under it, represent the limits of agreements. **(A)** Measurements at rest: the difference in the means (bias) was −0.26 L/min (95% CI, −0.62 to 0.09 L/min), with the lower and upper limits of the agreement being −1.92 and 1.39 L/min, respectively. **(B)** Measurements at end-exercise: bias was 0.29 L/min (95% CI, −0.08 to 0.67 L/min), with the lower and upper limits of the agreement being −1.25 and 1.83 L/min, respectively.

Resting values: The distribution of CO values is illustrated in Figure 2A. Bland-Altman analysis showed a bias of −0.26 L/min (95% CI, −0.62 to 0.09 L/min) and lower and upper limits of agreement of −1.92 and 1.39 L/min, respectively (Figure 3A). The percentage error was 28.6%. The coefficient of variation and the repeatability coefficient were 25.3% and 2.03 L for DF, respectively, 25.9% and <0.01 L for DFp.

End-exercise values: The distribution of CO values is illustrated in Figure 2B. The bias was 0.29 L/min (95% CI, −0.08 to 0.67 L/min). Likewise, the limits of agreement showed no drastic changes, with the lower limit at −1.25 L/min and the upper limit at 1.83 L/min (Figure 3B). The percentage error decreased to 16.1%. Compared to rest measurements, the coefficient of variation was increased marginally while the repeatability coefficient was completely inversed, being 0.99 L for DF and 1.72 L for DFp.

Resting and end-exercise values: Including both resting and end-exercise measurements, the bias showed no noteworthy change [0.01 L/min (95% CI, −0.35 to 0.37 L/min)]. Similarly, limits of the agreement remained comparable (−1.67 to 1.69 L/min). The percentage error was 22.3%, which equals a decrease of 6.3% compared to the resting value.

### DF Using Blood Gas vs. Intermittent TD

The average COs of both methods at rest, end-exercise, and both combined, and further statistical parameters are summarized in Table 2. The regression equation for TD and DF, i.e., rest and end-exercise, had a slope of 1.07 with an intercept of −0.20 L/min and an R^2^ of 0.72 (Figure 1B). However, neither normality (*p* < 0.05) nor homoscedasticity (*p* < 0.05) were met.

**Figure 4:**
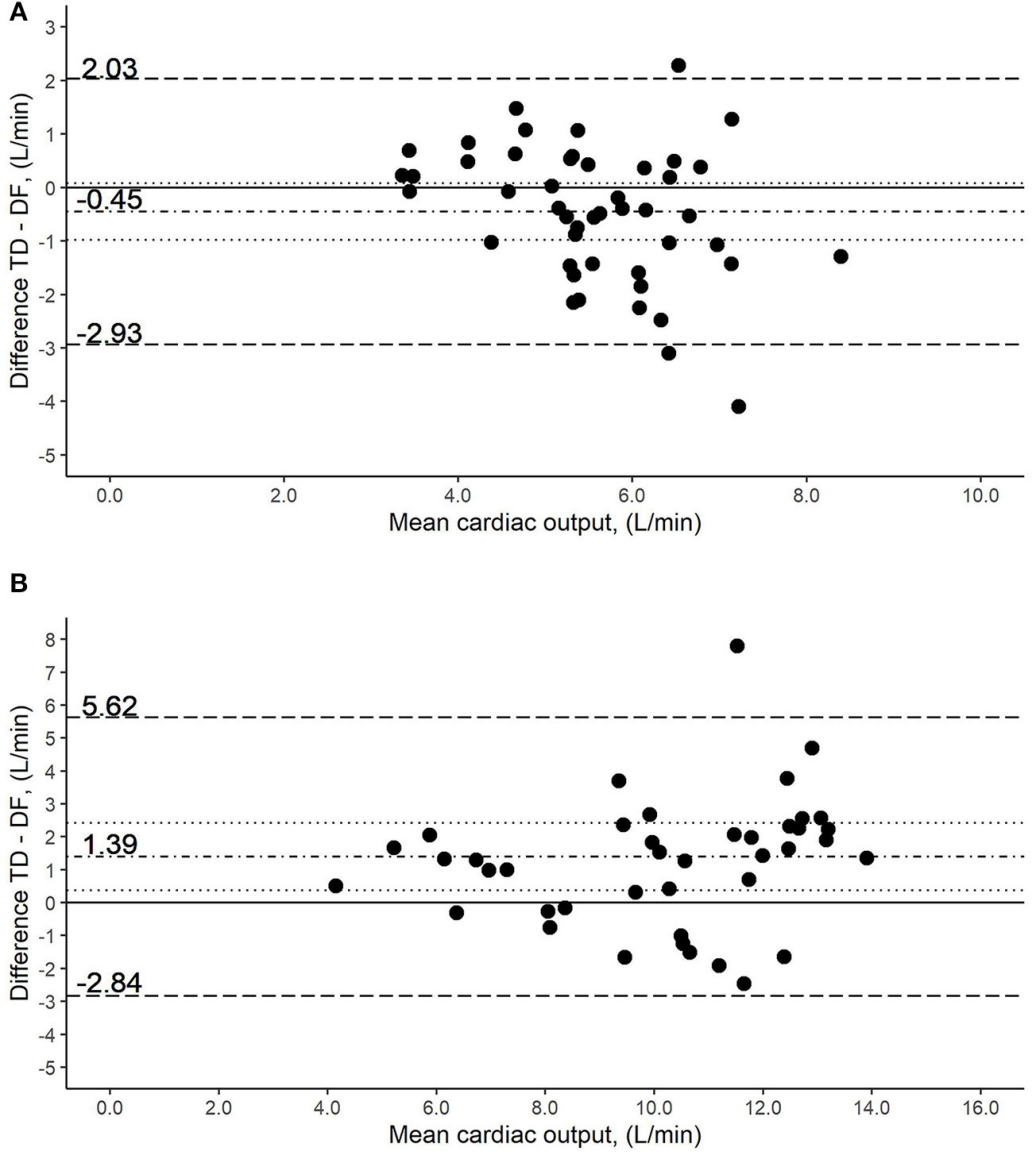
Bland-Altman plot of the comparison of cardiac output (CO) measurement methods by intermittent thermodilution (TD) vs. direct Fick using blood gases (DF). Y-axis was calculated as the mean of the two methods being compared. The solid line represents no difference in the means by the two methods. The dot-dashed line around zero represents the measured difference of the means, while the dotted lines stand for the 95% CI. The broken lines above and under it, represent the limits of agreements. **(A)** Measurements at rest: the difference in the means (bias) was −0.45 L/min (95% CI, −0.98 to 0.08 L/min), with the lower and upper limits of the agreement being −2.93 and 2.03 L/min, respectively. **(B)** Measurements at end-exercise: bias was 1.39 L/min (95% CI, 0.37–2.42 L/min), with the lower and upper limits of the agreement being −2.84 and 5.62 L/min, respectively.

Resting values: The distribution of CO values is illustrated in Figure 2C. The bias was −0.45 L/min (95% CI, −0.98 to 0.08 L/min). The limits of agreement were −2.93 and 2.03 L/min, respectively (Figure 4A). The percentage error was 44.6%, which equals an increase of 16.0% compared to DF vs. DFp. The coefficient of variation and the repeatability coefficient for TD were lower than that for DF.

Exercise values: The distribution of CO values is illustrated in Figure 2D. A bias significantly different from zero was detected with 1.39 L/min (95% CI, 0.37–2.42 L/min. The lower and upper limits of agreement were −2.84 and 5.62 L/min, respectively (Figure 4B). The percentage error was 41.6%, which equals a decrease of 3.0% compared to resting measurements. The coefficient of variation and the coefficient of repeatability were increased and were comparable to that of DF.

Resting and exercise values: Including both rest and end-exercise, the bias was 0.36 L/min (95% CI, −0.42 to 1.13 L/min). The limits of agreement were −3.28 and 4.00 L/min, respectively. The percentage error was 47.2%. Both, the coefficient of variation and the repeatability coefficient were increased more in relation to their counterparts of DF.

## Discussion

In this first study, comparing a modified DFp to the DF using arterial and mixed-venous blood gases, we found that the accuracy and precision to measure the CO by the modified DFp were within acceptable boundaries over a wide range of CO values. These results are of high interest, as simultaneous assessments of mPAP and CO are not possible when blood gases have to be sampled from the pulmonary artery through the RHC line, which limits PVR assessments during biologically unstable conditions such as exercise (18–20). The accuracy and precision to assess the CO by TD vs. the DF revealed lower agreements, which may limit its use, especially during incremental exercise.

Percentage error and limits of the agreement should be defined *a priori*, in the clinical context, and reported with repeatability assessments (21–23). Nevertheless, most comparison studies fail to report those statistical parameters and objective cut-offs for patients with PH are not established (24). Rather those studies quote the 30% percentage error as cut-off, as suggested by Critchley and Critchley (17) assessing TD vs. DF. In this work, we studied the accuracy and precision of CO assessment using DFp and put it into context to our predefined acceptance criteria over a wide range of values, which were obtained at rest and end-exercise in patients with PH. Thus, our acceptance criteria were based on clinical experience and the wide range of CO levels. In a review of CO comparison studies in patients with PH, the acceptance criteria were limits of agreement of ±1 L/min and a percentage error of 20%. However, those cut-offs were based on critical care and anesthesiology medicine, because studies on patients with PH were lacking (24). Since there is no established cut-off for the percentage error, we compared it to the widely used 30% limits mentioned above. In addition, we report the coefficient of variation and repeatability coefficient to allow correct interpretation of the Bland-Altman plot (21, 25). The coefficient of variation describes the contribution of a method to the overall agreement and precision of the two methods. A reference method with a higher coefficient of variation makes an interpretation of the Bland-Altman plot unreasonable (22). The repeatability coefficient is another statistical parameter, which helps to understand the contribution of a method to the lack of agreement (26). It reflects the variation in repeated measurements of the same method. However, it requires repeated measurements or “linked replicates” (16) to be taken into account, which a minority of CO comparison studies reports (24).

### DF Using Blood Gases vs. DF Using Pulse Oximetry

Our comparison between the DF and DFp methods showed no displacement of the regression line over the line of identity (Figure 1A), and the mean of the differences was not significantly different from zero (Figures 3A,B), which indicates that adequate accuracy/bias of DF is compared to DFp. Also, the percentage error and the limits of agreement were within predefined acceptance criteria of 30% and 2 L/min (Table 2).

At the end-exercise, the percentage error between DFp and DF was decreased drastically while the absolute limits of the agreement remained the same (Table 2), reflecting an even better precision for the DFp method under more strenuous work and therefore higher CO values. These end-exercise results may even better reflect the precision of DFp to DF, as in our study, setting arterial and mixed venous blood gases were sampled in the supine resting position, whereas VO_2_ was obtained starting 10–15 min after the patient was set in a stable semi-supine position. This might have influenced the limits of agreement between those two methods at rest. However, this time lag was inevitable to avoid too many blood samples.

Overall, the bias, limits of agreement, and percentage error between CO measurements by DFp and DF were within an acceptable range at rest and good at end-exercise measurements. The coefficient of variation remained approximately the same at rest, end-exercise, and both combined, with the reference method (DF) always showed a smaller value and allowed a rational conclusion of the Bland-Altman plot (22). It suggests that both methods contributed almost equally to agreement/precision in the comparison. Potential oximetry pitfalls, such as hypoperfusion, shivering, or movement artifacts during exercise, cannot be excluded having influenced the coefficient of variation (27, 28). The course of the repeatability coefficient and coefficient of variation were found slightly discordant, and the coefficient of repeatability varied during exercise. This may be explained by the crossover study design and our assumptions of interchangeability of the replicates between the three phases and the resulting long pauses between them. The DF as a gold standard method to assess CO requires simultaneous blood samples from an artery and the catheter-tip, which requires trained staff that should handle the samples without delay under avoidance of air admixture. Given the fact that DFp does not have these drawbacks and allows for a simultaneous mPAP and therefore pressure/flow measurement, it is a promising alternative to DF and maybe even less prone to errors in case of repetitive assessments during exercise (24). Studies compared other non-invasive methods for CO measurements, such as impedance cardiography to DF, which showed acceptable agreement and precision compared to DF or TD in patients with PH within a CO range of 3–7 L/min (29, 30). Further studies focused on the indirect Fick method to assess CO, which does not measure VO_2_ but assumes it from nomograms (31). These studies concluded that the LaFarge method was the most precise, but the indirect Fick method was associated with large errors and cannot be recommended (3, 32–36). However, those nomograms are not intended for exercise protocols and would possibly be associated with misdiagnosis of PH.

### DF Using Blood Gas vs. Intermittent TD

Thermodilution requires repetitive similar injections of cold saline, which needs time and precludes simultaneous measurement with mPAP, which may introduce bias to pressure/flow measurements in a biologically variable system, especially during exercise (37–39). Additionally, in comparison to DF, TD is more imprecise for lower CO values and when valvular regurgitation is present (40)—conditions that are frequent in patients with PH (9, 41). As simultaneous assessments are challenging, there have been only a few reports on the agreement between TD and DF in patients with PH, (36) especially during exercise.

The additionally performed CO measurement by intermittent TD did not reveal an acceptable overall agreement with DF during high CO levels, as obtained at the end-exercise. Neither limits of the agreement nor percentage errors were within predefined criteria (Table 2, Figures 4A,B). Although the present study revealed that the limits of agreement between TD and DF were relatively wide at rest, the regression line and its confidence interval showed no displacement above the line of identity over a broad range of CO values (Figure 1B). Furthermore, the coefficient of variation was lower than that of the reference method at rest, which renders the rational interpretation of the Bland-Altman plot questionable. A potential explanation for the discrepancies in precision and accuracy would include the response time of the measurement technique, which is aggravated by the time needed for triplicate cold saline injection. Additionally, the limitation of time lag as discussed in the comparison of DFp to DF may also have influenced the measurements.

Measurement of CO is even more challenging during stepwise incremental exercise, where steps are suggested to be at least 3 min to reach a steady state (42). Our study shows that agreement of TD to DF is lower at end-exercise than at rest, suggesting a systematic error at high CO values (43). Additionally, this finding indicates that patients with PH presumably need more than 3 min to reach a steady state. This would support the findings of Lador et al., who investigated “CO kinetics at the onset of exercise in patients with PH” and observed that up to 5 min are warranted to reach a steady state (44). Another study showed that mPAP/CO slopes calculated from CO by TD lead to significant overdiagnosis of exercise PH compared to CO measured by DF, (45) potentially related to exercise-induced right-to-left shunting, which is commonly found in patients with PH (46). However, in the present study, CO was rather overestimated by TD compared to DF at end-exercise (Figure 4B). Our and other studies' results thus suggest that accuracy and precision of the CO measurement during stepwise incremental exercise protocols by the TD method compared to the DF method is lower and thus particularly absolute CO values measured by this method must be interpreted with caution and in the context of clinical practice and research (47).

## Limitations

This study used repetitive CO measurements at rest and end-exercise obtained in the context of a randomized-controlled crossover trial studying the acute effect of acetazolamide in PH and was not specifically designed to compare CO measurements between DF, DFp, and TD, which may have influenced agreement and reproducibility in our setting. To assess the repeatability coefficient more precisely, we would suggest a study design with less time in-between the replicate measurements, because CO is biologically highly variable, reacting to slight changes of physical or emotional stress, body position, and other factors. We also did not compare the CO on different exercise levels between rest and end-exercise, as we did not draw arterial- and mixed venous blood at each exercise step judging repetitive blood sampling as unacceptable for patients.

## Conclusion

High-frequency repetitive CO measurements at rest and during stepwise incremental exercise by the modified DFp, using pulse oximetry at the catheter- and fingertip, reveal acceptable agreement with the golden standard DF over a broad range of CO values in patients with PH. Thus, this modified DFp method maybe a practicable method to assess CO simultaneously with the mPAP, especially during exercise, using everyday equipment, such as the Swan-Ganz catheter.

## Data Availability Statement

The raw data supporting the conclusions of this article will be made available by the authors, without undue reservation.

## Ethics Statement

The studies involving human participants were reviewed and approved by Kantonale Ethikkomission Zürich. The patients/participants provided their written informed consent to participate in this study.

## Author Contributions

SU is the guarantor of the paper. All authors meet criteria for authorship as recommended by the International Committee of Medical Journal Editors and contributed to the production of the final manuscript with revision for important intellectual content.

## Funding

This study was supported by the Swiss National Science Foundation (No. 32003B_146246/1) and Zurich lung.

## Conflict of Interest

None of the authors reports any conflict of interest in direct relation to this manuscript. SU reports grants from the Swiss National Science Foundation and Zurich Lung related to this work. She reports a grant from the Swiss Lung League, Janssen SA, Switzerland, Orpha Swiss, personal fees from Actelion SA, MSD, Orpha Swiss and Novartis, Switzerland, outside of the submitted work. The remaining authors declare that the research was conducted in the absence of any commercial or financial relationships that could be construed as a potential conflict of interest.

## Publisher's Note

All claims expressed in this article are solely those of the authors and do not necessarily represent those of their affiliated organizations, or those of the publisher, the editors and the reviewers. Any product that may be evaluated in this article, or claim that may be made by its manufacturer, is not guaranteed or endorsed by the publisher.
